# Exercise Training Protocols in Rabbits Applied in Cardiovascular Research

**DOI:** 10.3390/ani10081263

**Published:** 2020-07-24

**Authors:** Wilson M. Lozano, Germán Parra, Oscar J. Arias-Mutis, Manuel Zarzoso

**Affiliations:** 1 Department of Physiology, Universitat de València, 46010 Valencia, Spain; wilsonmauriciolozano@gmail.com (W.M.L.); german.parra@uv.es (G.P.); oscarariasphd@gmail.com (O.J.A.-M.); 2 INCLIVA, Instituto de Investigación Sanitaria, 46010 Valencia, Spain; 3 CIBERCV, Instituto de Salud Carlos III, 28029 Madrid, Spain; 4 Department of Physiotherapy, Universitat de València, 46010 Valencia, Spain

**Keywords:** exercise training, exercise protocols, rabbit, cardiovascular system

## Abstract

**Simple Summary:**

Several animal models have been used to understand the physiological adaptations produced by exercise training in the healthy and diseased cardiovascular system. Among those, the protocols for acute and chronic exercise in rabbits present several advantages compared to other large animal models. In addition, the rabbit model has important physiological similarities with humans. On the other hand, the design of the training protocol is a key factor to induce the physiological adaptations. Here, we review the different training protocols used in rabbits and the different physiological adaptations produced in the cardiovascular system, in normal and pathological conditions.

**Abstract:**

Rabbit exercise protocols allow for the evaluation of physiological and biomechanical changes and responses to episodes of acute or chronic exercise. The observed physiological changes are normal responses to stress, that is, adaptive responses to maintain or restore homeostasis after acute exercise. Indeed, the rabbit model is advantageous since (a) it has important physiological similarities in terms of the functioning of multiple organ systems, and can quickly induce alterations in pathophysiological conditions that resemble those of humans, and (b) it allows the implementation of a low-cost model in comparison with other large animals. When designing an exercise training protocol for rabbits, it is important to consider variables such as race, gender, age and, especially, training parameters such as volume, intensity, or rest, among others, to determine the outcome of the research. Therefore, the objective of this review is to identify and analyze exercise training protocols in rabbits in different experimental applications and the various physiological adaptations that are presented, with special focus in cardiovascular adaptations.

## 1. Introduction

Regular exercise has positive influences on multiple organ systems and can prevent or improve symptoms of several chronic illnesses, including cardiovascular disease (CVD). In both basic and applied research, efforts have been made to elucidate the benefits of physical exercise on the cardiovascular system, as well as the underlying physiological mechanisms. Studies have previously described endurance exercise training as a simple, low-cost, non-pharmacological measure to intervene in unhealthy habits and lifestyles, demonstrating feasibility, convenience, and practicality [1,2,3,4]. Various experimental animal models, including small (mice, rats, rabbits) and large (canine, sheep, pig) animals have been utilized to understand the mechanisms involved in the changes associated with physical activity. Furthermore, animal models allow for the evaluation of physiological adaptations involved in the interaction of particular pathological states, such as CVD or metabolic syndrome (MetS) [5,6,7].

The expected effects of interventions with exercise training depend largely on the animal model (species) chosen, type of exercise (continuous or intermittent), intensity (low, moderate, vigorous) and/or type of energetic pathway involved (aerobic, anaerobic), time of day (day, night), and the duration of the protocol (acute, chronic) [8]. Exercise training protocols are designed based on these variables. In the case of the protocols used in rabbits, there is no agreement or standardization regarding the optimal way of combining those variables and training modalities, as it happens in other animal models such as rodents [9,10], and there exists a great heterogeneity in the protocols used that makes difficult to compare the effects among the different studies. Training protocols generally use activities such as running on a treadmill, swimming, or voluntary wheel race [8,11].

Experimental rodent models have been widely used in exercise training studies, even though they have multiple limitations [1,9,12,13], especially in the context of studying cardiac electrophysiological remodeling. Indeed, the murine ventricle shows a short (approximately 50 ms), spike-shaped action potential, and substantial electrophysiological differences in repolarization due to the different expression of ion channels that carry the transmembrane currents that shape the action potential, especially the outward K^+^ currents (*I*_Kr_, and *I*_Ks_) [14]. In contrast, in the rabbit ventricular myocardium, the morphology of the action potential is spiked and dome-shaped, with the duration approximately 200–300 ms and repolarization mediated mainly by *I*_Kr_ and *I*_Ks_, similar to that seen in the human ventricle [15,16,17]. In addition, the rabbit model has been widely used to study sustained arrhythmias and ventricular fibrillation (VF) [18,19,20]. Moreover, rat and mice hearts have quantitatively different reliance on calcium-handling proteins when compared with larger animals and humans [21]. In addition, the rabbit model has important physiological similarities in terms of the functioning of multiple organ systems and can induce alterations in pathophysiological conditions that resembles that of humans [4,12]. For instance, it has been reported that rabbits fed a high-fat diet show hemodynamic and neurohumoral changes similar to those observed in obese humans [6]. As herbivorous animals, rabbits are susceptible to high-fat diets, and they have high baseline plasma lipid transfer protein (CETP) and low-density lipoprotein (LDL) profiles, similar to those in humans [22]. The similarity in lipoprotein metabolism contributes to their usefulness as a translational model for the study of pathologies with cardiovascular effects such as obesity, hypertension, atherosclerosis, and metabolic syndrome [23]. Certainly, last but not least, the rabbit model is advantageous as it allows low-cost implementation in comparison with other large animals, in terms of time of protocols, human and economic resources needed, and its affordability for chronic protocols and monitoring. 

Therefore, this review aims to identify and analyze the different exercise training protocols applied in experimental studies using rabbits, their characteristics, and their varying physiological adaptations, with special focus in the cardiovascular alterations in physiological and pathological conditions.

## 2. Exercise Training Protocols in Rabbits

When designing the exercise training plan for rabbits, it is important to respect the usual behavior of the species in order to prevent stressful stimuli during exercise [21]. The exercise protocols for rabbits widely vary depending on the objective of the research. In general, they allow for the evaluation of physiological and biomechanical changes in response to acute or chronic episodes of exercise, and thus, to investigate the effects derived from the type of exercise in the cardiovascular system. Indeed, several studies have shown that experimental rabbits can complete training protocols on a treadmill with intermittent or continuous running, applying different exercise intensity, duration, and rest [21,24].

Acute exercise is defined as a single episode of physical activity or exercise that is not repeated on a regular basis. Whereas, exercise training or chronic physical exercise is described as a series of regular episodes of physical exercise systematically repeated over several days and weeks. The effects of both acute and chronic exercise have been well characterized and reviewed under physiological conditions [25]. A wide range of exercise-induced cardiovascular changes in animal models resembles the behavioral and physiological effects seen in humans [25,26,27].

The physiological changes observed are normal responses to stress, namely, adaptive responses to maintain or restore homeostasis after acute exercise. In the case of chronic exercise protocols, in addition to observing adaptive physiological responses, changes related to prolonged stress that can significantly impact the animal’s condition have also been taken into account [1,7,21].

### 2.1. Studies on the Effects of Acute Exercise in Rabbits

During exercise, the autonomic nervous system (ANS) rapidly adjusts to the various systems of the body in response to increased metabolic demand in stressful situations. The immediate responses are thought to be due to the execution of acute exercise and, initially, are reflected in the cardiovascular system, as this is the first to respond to the increased metabolic demand. In this sense, variations such as increased heart rate, increased blood pressure, redistribution of blood flow to different organs, and consequently, increased oxygen consumption occurs [21,28]. Table 1 summarizes the available protocols related to the effects of acute exercise.

Laboratory rabbits are able to run at different speeds continuously or intermittently on a motor-driven treadmill with adjustable inclination (0–15°). Meng and Pierce [24] determined the ability of rabbits to run continuously or intermittently at relatively high speeds on a treadmill machine. At the same time, they investigated the metabolic and physiological responses of rabbits to acute episodes of exercise. Before commencing the exercise protocol, the animals went through an adaptation period of 3 days per week for 3 weeks. During the first week, the rabbits ran for 1–3 min at 6 m/min, and by the third week, the rabbits were reaching a speed of 20 m/min during 2 min of continuous running. When the rabbits were moved on to the experimental phase of the intervention protocol, the continuous exercise group ran at a speed of 15–20 m/min for 3 min, 5 min, or until fatigue. On the other hand, the intermittent exercise group reached speeds between 40 and 50 m/min for 30 s followed by an equal period of time for rest, or maximum speeds up to 70 m/min for 15 s with double the time of rest. A 2° inclination was used throughout the experiment [24].

On the other hand, O’Hagan et al. [28] used a period of familiarization with the treadmill 1 or 2 times per week for 2–5 weeks, at a speed ranging from 7 to 12 m/min without inclination for 2–5 min. In another study from their group, the familiarization period was extended from 5 to 19 weeks, with the speed increasing to between 10 and 12 m/min and a 20% grade inclination [29]. With respect to the experimental phase, this study used two different protocols. In the first protocol, the animals ran at a speed of 10–12 m/min for 2–5 min. In the second protocol, the group ran for 5 min at a speed of 7–10 m/min, with a rest period of 30–45 min between protocols. At the end of the exercise, the animals showed no signs of fatigue. In addition, each exercise series had a rest period of 2 min, 2 or 5 min of exercise, and recovery for up to 10 min [28,29]. In a similar study, Mueller et al. [30] used 3 protocols with progressive increases of intensity including 7 m/min for 5 min, 12 m/min for 2 min, and 15 m/min at 17% (10°) inclination until exhaustion.

Gaustad et al. [21] proposed an optimal protocol to evaluate the maximum oxygen consumption (VO_2_ max), where VO_2_ max was measured in rabbits at various inclinations (0, 5, 10, 15, and 20°). Therefore, the increase in intensity could be approached taking into account the increase in VO_2_. Each rabbit had a 10 min warm-up at 40–50% of the VO_2_ max prior to the protocol. The speed of the treadmill was increased by 0.03 m/s every 2 min until exhaustion. During the exercise protocol, submaximal VO_2_ values were estimated; the rabbits ran the five inclinations twice in random order. Before the submaximal test, the rabbits warmed up for 10 min at 40–50% of their VO_2_ max and then ran for 4 min at intensities between 50 and 75% of their VO_2_ max.

### 2.2. Protocols for Exercise Training in Rabbits

It has been demonstrated that exercise is effective in preventing the initiation or progression of cardiovascular alterations through lifestyle modification [31] and through effects directly derived from physical exercise and training. Therefore, it is suggested that long-term exercise protocols should be used as a therapeutic intervention in subjects with conditions such as chronic heart failure, hypertension, atherosclerosis, and MetS [31,32,33].

In general, the protocols for exercise training in rabbits (Table 2) begin with an adaptation phase for the animal, called the familiarization period. This period varies from 3 days [34], 4 days [35,36], or 1 week [31,32,33,37,38], to several weeks [24,29,39,40,41]. These adaptation periods should be constantly monitored (observation and accompaniment) to avoid the animals running inappropriately or stopping during the execution of the training protocol [35].

**Figure 1:**
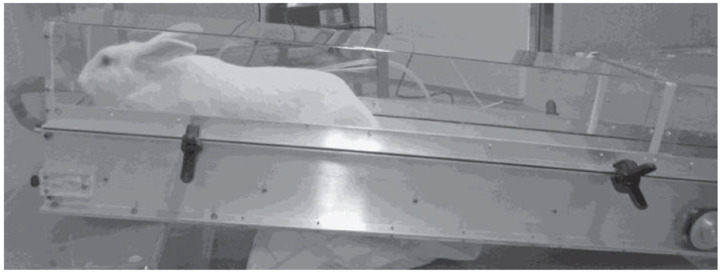
Adjustable speed and tilt treadmill. Adapted from Gaustad et al. [21].

In the application of exercise training protocols, the most used device is the electrically adjustable speed treadmill (Figure 1). However, there are also reports of the use of voluntary running, either using a mechanical drive treadmill [38,42] or a rotating wheel (motor driven wheel) [32].

#### 2.2.1. Treadmill Training Protocols

(a) Continuous training protocols

Continuous exercise protocols have been reported using treadmill inclination (from 5 to 12%) at speeds between 2.5 and 3 km/h, 20 min a day, 5 days per week for over 16 weeks [40]. Other protocols have used 4–5 weeks of continuous running training without treadmill inclination at a speed of 13 m/min, for 20 min, preceded by 5 min of warm-up and 5 min of cool-down at a speed of 8 m/min [57].

Similarly, several authors have proposed a 40 min protocol, with 5 min of warm-up (5 m/min) followed by 30 min of continuous running at a speed of 15–18 m/min, ending with 5 min of cool-down, 6 days a week [33,50,56]. The same training time has been used by Chen et al. [41] 5 days per week for 4 weeks at a constant speed of 1 km/h. 

Another long-duration continuous training modality included progressive increases in intensity. Carroll et al. [46,51] used exercise training in rabbits after acute myocardial infarction. Their protocol consisted of continuous running on a treadmill for 12 weeks, 5 days per week, and with progressive workloads that were adjusted weekly at a speed of 16.1–21.4 m/min, with a maximum duration of 30 min per session. When the workload exceeded 30 min, the work was divided into 2 sessions of continuous running separated by at least 1–3 h of rest. The duration of exercise during the first week was 7–8 min and increased progressively to 50–60 min until week 12.

Furthermore, Wang et al. [34] carried out a progressive protocol of continuous running 6 days per week, for 4 weeks, at a speed of 10 m/min for the first 3 days of training, 15 m/min from the fourth to the sixth day of training, and 20 m/min from the seventh day until completing the 4 weeks of training. Other studies applied a 12-week training protocol, 5 days per week, in which each exercise session lasted up to 60 min. Training started with continuous running on a treadmill at a speed of 10 m/min for 1 min, followed by a speed progression of 3 m/min every minute until the rabbit was exhausted [39,54,55]. 

Similarly, Hexenberg et al. [45] conducted a protocol of progressive endurance training for 10 weeks. The rabbits trained 5 days a week on a treadmill with exercise periods increasing weekly by 5 to 10 min until the last week of training. The speed was also increased from 0.5 to 1.2 km/h.

On the other hand, Yang et al. [48] proposed an exercise protocol with an intensity of approximately 70% of the maximum heart rate during exercise. The first week of continuous running involved the animals exercising at a speed of 0.9 km/h for 10 min. In the following weeks, the exercise increased by 5 to 10 min each day until 60 min per day was completed, for 5 days per week during a total of 8 weeks [37]. The same running speed was used in another study, but the training protocol was applied 5 days per week for 6 weeks, with the training period increasing from 30 to 40 min per day [31].

(b) Interval training protocols

Several authors have proposed the implementation of intervallic running protocols on adjustable speed treadmills. Some intervallic exercise protocols include 5 sessions per week, for 6 weeks. Each session is divided into 6 exercise intervals. The animals run for 4 min at a speed, depending on the study, of 0.33 m/s [21,36] or 0.5 m/s [35], separated by a 1 min rest between each run.

Becker et al. [52] carried out an extensive intervallic exercise protocol consisting of 4 min of warm-up at a speed of 11 m/min, followed by 26 min of continuous exercise divided into four intervals: 6 min at 16 m/min, 10 min at 27 m/min, 6 min at 16 m/min, and ending with a 4-min period at 11 m/min for the animals’ return to rest. The total exercise time per session was 30 min. Exercise sessions were performed once a day, 5 days per week, for 8 consecutive weeks. 

#### 2.2.2. Voluntary Training Protocols

Few studies have carried out experimental protocols of continuous voluntary running of rabbits on a treadmill. In the mid-1980s, Videman et al. [43] and Friman et al. [44] employed a protocol in which rabbits either ran uphill on a treadmill at a 20° slope or ran on a surface without a slope, at a speed of 0.15 m/s, 3 times a day at will and until exhaustion. On days 1, 5, and 30 of the experiment, the median daily running distance was 600 m. 

Subsequently, Szabo et al. [38,42]. applied a protocol that included 2 voluntary sessions until exhaustion, twice a day. The animals managed to reach a speed of 3–9 m/s, to complete distances of 1.2–1.6 km per day, without any stimulus or use of electric current pulses during the session [38,39]. Szabo et al. [42] also used moments to control the speed of the treadmill by oscillating between 3–6 m/s to avoid possible injuries derived from the automatic stimulation of the running machine. At this speed, the animals reached distances between 0.6–0.8 km per day. 

#### 2.2.3. Training Protocols on Rotating Wheel

Exercise protocols in small rodents usually involve treadmills, swimming, and/or a rotating wheel. The latter has been used for the study of chronic exercise in various rodent species and, compared to other types of exercise, requires voluntariness for the propulsion of the device. Therefore, in this case, it resulted in reduced stress for the animal [26]. 

Although there is little evidence available, with reference to the use of a rotating wheel in medium-sized animals such as rabbits, Liu et al. [32,47] reported the use of a rotating wheel powered by a motor based on a proprietary design. An exercise training protocol was applied to the wheel with a gradual increase in speed to 12–18 m/min, for a total time of 40 min (warm-up 5 min, continuous exercise 30 min, and cooling down 5 min), 6 days per week for 4 weeks.

## 3. Special Considerations When Applying Rabbit Training Protocols

It has been demonstrated in short-term exercise protocols that rabbits can learn to run continuously at different speeds (slow, moderate, and relatively high) on a controlled treadmill. Observations indicated that rabbits are better suited to run intermittently at higher speeds than continuously at lower speeds. Therefore, rabbits used to be more motivated to run intermittently than continuously on treadmills with adjustable speed [24]. This behavior is thought to be due to the habits of this animal in the wild. Rabbits are prey animals and, when stalked by a predator, they will often stop running to camouflage themselves, which is an important aspect for handling rabbits in exercise protocols. It is also a priority that the animal is not stimulated with a stressful environment/stimulus during the exercise [23].

On the other hand, in laboratory rabbits, there are conflicting reports about their willingness to run. Studies conducted by Jover et al. [58] and Faris et al. [59] reported that rabbits rarely ran at a moderate pace of 13 m/min for more than 1–2 min. They also described that laboratory rabbits had a limited capacity for aerobic exercise because they are outside their natural environment [21,58,59]. In spite of this, studies have shown that rabbits can perform voluntary aerobic exercise on a treadmill continuously at a moderate speed [38,49]. 

In addition, during the period of adaptation to the protocol, the rabbits can learn to run on a treadmill at moderate and high speeds, in which they tend to change the typical jump pattern for a continuous gallop pattern. In this setting, low speeds have been seen to promote the jump pattern [24]. In this regard, the use of young or young adult rabbits has been recommended in these types of studies since they are more receptive and motivated to run on a treadmill, without the need for stimulation by means of electrical shocks. This practice, even though is commonly used in other species, is not recommended in the laboratory rabbit given that they are very sensitive and easily stressed animals. Some authors recommend the use of a brush or small wooden sticks to motivate the animals when they rest for more than 2–3 s in the back of the treadmill [24].

O’Hagan et al. [28] estimated the running speed of rabbits in relation to aerobic capacity. In their study, they observed that when the rabbits ran at a speed of 12 m/min for 2 min, they reached an intensity of approximately 92% of the estimated aerobic capacity. Furthermore, this study determined that 84% of the estimated aerobic capacity was achieved by reaching a speed of 7 m/min during a 5 min period.

On the other hand, less attention has been paid to aspects such as the gender and breed of animals used in exercise protocols, with most studies using New Zealand-bred male rabbits. In fact, few studies used California rabbits [43,44] and Pannon rabbits [42,49], compared to the vast majority of studies and protocols using New Zealand white rabbits. [24,33,35,36,41,46,47,52,54,56,57]. 

With regard to the gender of the animal, none of the studies describe the reasons for the choice of gender in the experimental models. Despite this, predominantly male rabbits are used [19,33,34,47,48,50,52,56,57,60], in contrast to the lesser use of females in the studies [21,24,46,51], and even less studies using rabbits of both genders [39,54,55].

## 4. Effects of Acute Exercise and Training on Rabbits and Clinical Utility

Physical training in rabbits has been used in multiple studies to assess both the immediate response to stress and the long-term adaptations produced by metabolic stress, induced by the systematic repetition of a series of exercises at a given intensity and time [1,9,13]. Even though the rabbit experimental model has many similarities with human physiology and pathophysiology, it is not the most widely used experimental model in the literature when using physical training protocols in basic studies [1,9,13]. In this section we analyze the most common responses to physical exercise and training.

One of the markers of the “trained state”, as it happens with humans, is the decrease in basal and intrinsic heart rate, which has been assessed in some studies [19,40,45,50]. Other studies report increases in muscle mass of the gastrocnemius, soleus, or left ventricle as a training parameter [45,46]. The measurement of the enzyme citrate synthase activity is also a common marker for the evaluation of the efficacy of the training protocol [31,33,37,53]. The decrease in plasma lactate concentration during and after exercise is used as well [21,24,38]. Regarding training evaluation, Gaustad et al. [21] presented a protocol in rabbits that could be used to determine, adjust individual training intensities, and evaluate the training protocol with the “gold standard” used in humans (VO_2_max). They found, as it happens in humans, a linear relationship among VO_2_, heart rate, and lactate concentration when increasing exercise intensity.

The activity of the sympathetic nervous system has been also a susceptible parameter to evaluation, inducing acute responses resulting from exercise. For example, the response of peripheral vasoconstrictive signaling has been studied in organs such as the kidney [28]. Since, under resting conditions, the kidney receives a very high percentage of cardiac output, it is not surprising that during exercise the opposite occurs due to the redistribution of arterial blood flow mediated by the renal sympathetic response [29,30]. On the other hand, it has been demonstrated that continuous running with progressive intensity protected the kidney of healthy rabbits, by enhancing the renal endothelium-dependent and -independent vascular reactivity. Furthermore, it improved the negative effects caused by acute exposure to moderate levels of glycemia in rabbits with induced type 2 diabetes mellitus [39,54].

The findings from the study by Mueller et al. [30] demonstrated decreased renal blood flow due to peripheral vasoconstriction from the beginning of exercise, and this perfusion was maintained throughout the training. In contrast, no initial vasoconstriction was observed in the denervated kidney, only a slow vasoconstrictive response was evident as exercise continued. This suggested that circulating catecholamines may contribute to the vasoconstrictive response during exercise, reinforcing the principal role of the ANS in the immediate response to metabolic stress. These findings reinforced the results of O’Hangan et al. [29], who evaluated the chemical reflex response resulting from a period of hypoxia, induced by exercise dynamics.

In addition, studies using long-term exercise protocols showed a significant benefit of exercise in relation to renal sympathetic response, blood pressure baroreflex control, and cardiac function, with significant vagal contribution during the resting state [32,47,61]. Li et al. [33] demonstrated that chronic exercise normalized the exaggerated response of the peripheral chemical reflex and the chemoreceptor sensitivity of the carotid body in rabbits with induced chronic heart failure, alongside preventing alterations to the angiotensin II type 1 receptor. Therefore, exercise reduced sympathetic activity and plasma catecholamines at rest, and improved the sensitivity of the cardiopulmonary reflex in rabbits with heart failure [50].

Meanwhile, the implementation of intermittent running exercise protocols on a treadmill has made it possible to evaluate the effects of exercise on some of the electrophysiological properties in the isolated rabbit heart. An intrinsic depression of the automatism of the sinus node and atrial-ventricular conduction has been demonstrated, as it happens in humans, with relatively short chronic exercise protocols [35]. In addition, increased ventricular refractoriness was demonstrated by prolonging the functional and effective ventricular refractory periods, and decreasing the dominant frequency of ventricular fibrillation. These modifications may be protective against the production and maintenance of reentrant arrhythmias and should be considered given the role of aerobic exercise as a non-pharmacological intervention against sudden cardiac death [19]. Similarly, progressive-intensity exercise (0.5–1.2 km/h) reduced the resting heart rate and increased the systolic volume [45].

Long-term protocols, including continuous running, have been shown to cause enlargement of the left ventricular cavity, changes in heart rate and heart rate variability parameters, indicating increased vagal tone [40]. These are the findings that can be seen in elite athletes in disciplines where aerobic endurance is predominant. The decrease in heart rate favors prolonged repolarization and electrophysiological ventricular homogeneity [40]. In addition, Gao et al. [56] concluded that exercise normalized the sympathetic response and the baroreflex mechanism in rabbits with chronic heart failure, accompanied by positive regulation of superoxide dismutase expression, thus improving the mechanisms of antioxidant pathways and prooxidant suppression in the ventral-lateral region of the spinal cord.

Moreover, Hexeberg et al. [45] reported that physiological hypertrophy was not observed in rabbits. However, they indicated the presence of structural remodeling of the myocardium by measuring the slope of the pressure–volume ratio at the end of diastole in trained animals. After 10 weeks of progressive exercise training, the slopes for the longitudinal segments of the left ventricle were less pronounced than in control rabbits, although shortening of the segments was similar. These findings may suggest that the myocardium has been structurally remodeled and the increase in contractile reserve can be recruited during adrenergic stimulation in the trained group.

On the other hand, experimental models of voluntary running in rabbits revealed that 4-week submaximal aerobic exercise, showed a significant decrease in plasma lactate dehydrogenase concentration. Regarding the profile of muscle fatty acids, exercise increased the proportion of oleic acid and significantly decreased the level of stearic acid and arachidonic acid. Therefore, these changes were attributed to regular exercise without subjecting the animal to stressful stimuli during involuntary training [38]. Meng and Pierce [24] also reported a 50% decrease in plasma triglycerides, and an increase in both creatine phosphokinase (CPK) and muscle glycogen after continuous and interval training.

Chen et al. [41] used an experimental model of acute myocardial infarction induced in New Zealand white rabbits. They observed that under these conditions, exercise training did not improve antioxidant capacity, but despite this, there was evidence of improvement in left ventricular heart function, promotion of autophagic function, and increased utilization of fatty acids; the latter two factors may have contributed to the improvement of heart function.

In addition, the implementation of exercise training protocols during the development of obesity in rabbits was associated with a decrease in diastolic blood pressure compared to sedentary obese rabbits. Exercise training did not alter the plasma levels of lipids or hormones (insulin, cortisol, renin, and aldosterone) in obese rabbits. These results suggested that training, in the absence of alterations in body weight, is an effective tool for blood pressure control over other treatments, which has the potential to be used for the control of other cardiovascular risk factors [46,51]. In addition, it significantly reversed the deleterious effects of a high-fat and cholesterol diet [37] on vascular functions at early stages (2 weeks), and reduced pro-inflammatory changes after 4 weeks [31], thereby reducing the progression of atherosclerosis [53].

## *5.* Conclusions

There is a great variety of exercise training protocols in rabbits which can be used to model the cardiovascular adaptations to training in humans. These experimental models of exercise training in rabbits have allowed for the investigation of the physiological effect of training, and in different pathologies with cardiovascular involvement, and their possible underlying mechanisms. Indeed, it has been found that the experimental rabbit model, in short- and long-term exercise scenarios, easily presents physiological adaptations similar to those described in humans. Furthermore, rabbit models represent an intermediate compromise between large animal and smaller (rodent) animal models, which makes it possible to carry out diverse research protocols with minimal personnel, maintenance, and resources.

However, from the analysis of the protocols, we have observed that there is a great heterogeneity in the literature and a lack of consensus regarding a process of design and validation of the training protocols that allows us to define or to standardize a protocol of exercise in rabbits. In this sense, this makes the determination, validity, and reproducibility of the evaluation of the aerobic fitness in rabbits and its associated changes very challenging. With the great potential of the rabbit as an experimental model that mimics human physiology, future studies would be required to develop reliable and valid protocols based on previous evaluation of the animal condition against the “gold standard” used in humans, the VO_2_ max, and subsequent design of a protocol that manages to optimally combine the different elements of the design (intensity, duration, volume, and rest).

## Figures and Tables

**Table 1:** Protocols for acute exercise in rabbits.

Author			Exercise Protocol					
	Breed	Age	Adaptation	Equipment	Inclination	Type	Speed	Duration
Meng et al. [24]	NZW	7 weeks old	3 weeks	Treadmill	2°	Continuous	15–20 m/min	3, 5 min, or until exhausted
						Intermittent	Moderate 40–50 m/min or high 70 m/min	30 s of moderate work followed by 30 s of rest or 15 s of high work followed by 30 s of rest.
O‘Hagan et al. [28]	NZW	NR	2–5 weeks	Treadmill	0°	Intermittent	7 m/min	5 min one or two bouts, 30 min of rest between bouts
					0°	Intermittent	12 m/min	2 min one or two bouts, 30 min of rest between bouts
O‘Hagan et al. [29]	NZW	NR	5 weeks once or twice per week	Treadmill	12°	Intermittent	10–12 m/min	Two 3–5 min bouts, 45 min of rest between bouts
Mueller et al. [30]	NZW	NR	1–2 times a week	Treadmill	10°	Continuous	15 m/min	One or two 2–5 min bouts
Gaustad et al. [21]	NZW	12 weeks old	NR	Treadmill	0°	Continuous	1.8 m/min every 2 min until exhaustion	4 min to exhaustion with rest of at least 24 min
					5°			
					10°			
					15°			
					20°			

The intensity scale was adapted to m/min. NZW, New Zealand White; NR, Not reported.

**Table 2:** Protocols of chronic exercise training in rabbits.

Author			Exercise Protocol					
	Breed	Age	Adaptation	Equipment	Intensity	Duration	Frequency	Period
Videman et al. [43]	Californian	39 weeks old	NR	Treadmill	9 m/min with 20° of inclination	Until exhaustion	3 times/day	30 days
Friman et al. [44]	NR	NR	NR	Treadmill	9 m/min with 20° of inclination	Until exhaustion	3 times/day	30 days
Hexeberg et al. [45]	NR	NR	NR	Treadmill	8.3–20 m/min	15–60 min/day	5 days/week	10 weeks
Carroll et al. [46]	NZW	15–17 weeks old	1 week	Treadmill	16.1–21.4 m/min	7–8 min until 50–60 min/day at the last weeks	5 days/week	12 weeks
Szabó et al. [42]	Pannon White	NR	1 week	Treadmill	3–6 m/min	Voluntary until complete 0.6–0.8 km/day	2 times/day	8 weeks
Liu et al. [47]	NZW	NR	1 week	Wheel	15–18 m/min	40 min/day	6 days/week	4 weeks
Such et al. [35]	NZW	NR	4 days	Treadmill	30 m/min	30 min/day	5 days/week	6 weeks
Jen et al. [37]	NZW	NR	1 week	Treadmill	14.7 m/min	10 until 60 min/day at the last weeks	5 days/week	8 weeks
Yang et al. [48]	NZW	NR	1 week	Treadmill	14.7 m/min	5–10 min until 60 min/day70% of MEC	5 days/week	8 weeks
Szabó et al. [49]	Pannon White	4 weeks old	1 week	Treadmill	3–9 m/min	Voluntary until complete 1.2–1.6 km/day	2 times/day	4 weeks
Yang et al. [31]	NZW	NR	1 week	Treadmill	14.7 m/min	30–40 min/day	5 days/week	6 weeks
Pliquett et al. [50]	NZW	NR	NR	Treadmill	18–20 m/min	40 min/day	6 days/week	3 weeks
Carroll et al. [51]	NZW	15–17 weeks old	NR	Treadmill	16.1–21.4 m/min	30 min until 50–60 min at 10 weeks	5 days/week	12 weeks
Becker et al. [52]	NZW	NR	NR	Treadmill	11–27 m/min	30 min/day	5 days/week	8 weeks
De Moraes et al. [39]	NZW	NR	2 weeks	Treadmill	18 m/min	60 min/day	5 days/week	12 weeks
Jen et al. [53]	NZW	NR	NR	Treadmill	14.7 m/min	40 min/day	5 day/week	6 weeks
De Moraes et al. [54]	NZW	NR	2 weeks	Treadmill	18 m/min	60 min/day	5 days/week	12 weeks
De Moraes et al. [55]	NZW	NR	2 weeks	Treadmill	18 m/min	60 min/day	5 day/week	12 weeks
Gao et al. [56]	NZW	NR	NR	Treadmill	15–18 m/min	40 min/day	6 days/week	4 weeks
Li et al. [33]	NZW	NR	1 week	Treadmill	15–18 m/min	30–40 min/day	6 days/week	5 weeks
Chen et al. [41]	NZW	NR	2 weeks	Treadmill	8.3–16.7 m/min	10–30 min/day	5 days/week	4 weeks
Zarzoso et al. [19]	NZW	NR	4 days	Treadmill	20 m/min	30 min/day	5 days/week	6 weeks
Marcus et al. [57]	NZW	NR	NR	Treadmill	8–13 m/min	30 min/day	5 days/week	5 weeks
Wang et al. [34]	NZW	11–12 weeks old	3 days	Treadmill	10–20 m/min	20 min/day	6 days/week	4 weeks
Polyák et al. [40]	NZW	47 weeks old	2 weeks	Treadmill	4.2–5 m/min with 3–7° of inclination	20 min/day	5 days/week	16 weeks
Marchio et al. [36]	NZW	NR	4 days	Treadmill	20 m/min	30 min/day	5 days/week	6 weeks

The intensity scale was adapted to m/min. NZW, New Zealand White; MEC, Maximal Exercise Capacity, NR, Not reported.
